# Comparative study on clinical outcomes and cost-effectiveness of chronic subdural hematomas treated by middle meningeal artery embolization and conventional treatment: a national cross-sectional study

**DOI:** 10.1097/JS9.0000000000000699

**Published:** 2023-10-12

**Authors:** Xin Tong, Xiaopeng Xue, Aihua Liu, Peng Qi

**Affiliations:** a Beijing Neurosurgical Institute, Beijing Tiantan Hospital, Capital Medical University; b Department of Neurosurgery, Beijing Hospital, National Center of Gerontology; Institute of Geriatric Medicine, Chinese Academy of Medical Sciences, Beijing, People’s Republic of China

**Keywords:** chronic subdural hematomas, conventional treatment, cost-effectiveness, middle meningeal artery embolization, propensity score matching

## Abstract

**Background:**

The authors compared the efficacy and cost-effectiveness of middle meningeal artery embolization (MMAE) and conventional treatment for chronic subdural hematomas (cSDH).

**Methods:**

The Nationwide Readmissions Database of 9963 patients (27.2% women) with cSDH between 2016 and 2020 was analyzed. Finally, 9532 patients were included (95.7%, treated conventionally; 4.3%, treated with MMAE). Baseline demographics, comorbidities, adverse events, treatment strategies, and outcomes were compared between patients treated with MMAE and conventional treatment. After propensity score matching, the authors compared primary outcomes, including the 90-day treatment rate, functional outcome, length of hospital stays, and cost. A Markov model estimated lifetime costs and quality-adjusted life years (QALYs) associated with different treatments. The incremental cost-effectiveness ratio (ICER) was calculated to evaluate the base-case scenario. One-way, two-way, and probabilistic sensitivity analyses were performed to evaluate the uncertainty in the results.

**Results:**

After propensity score matching, MMAE had a lower 90-day retreatment rate (2.6 vs. 9.0%, *P*=0.001), shorter length of hospital stays (4.61±6.19 vs. 5.73±5.76 days, *P*=0.037), similar functional outcomes compared (favorable outcomes, 80.9 vs. 74.8%, *P*=0.224) but higher costs ($119 757.71±90 378.70 vs. $75 745.55±100 701.28, *P*<0.001) with conventional treatment. MMAE was associated with an additional cost of US$19 280.0 with additional QALY of 1.3. Its ICER was US$15199.8/QALY.

**Conclusion:**

MMAE is more effective in treating cSDH than conventional treatment. Based on real-world data, though MMAE incurs higher overall costs, the Markov model showed it to be cost-effective compared to conventional treatment under the American healthcare system. These comparative and economic analyses further support the consideration of a paradigm shift in cSDH treatment.

## Introduction

Highlights

- Middle meningeal artery embolization (MMAE) for chronic subdural hematoma leads to lower retreatment rates and shorter hospital stays.
- MMAE improves functional outcomes similar to conventional treatment.
- Patients with MMAE are more cost-effective than conventional treatment.

Chronic subdural hematoma (cSDH) is defined as the subdural collection of blood over a period of greater than 3 weeks. Its annual incidence rate is ~13.5 per 100 000 individuals, reaching up to ~58.1 per 100 000 individuals in patients aged greater than 65 years^1^. Additionally, its incidence is expected to increase^2^. The 1-year mortality rate of cSDH is as high as 30%^3^. Unfortunately, there are currently no established guidelines for cSDH management^3,4^. Three primary conventional treatment techniques are used: twist drill craniotomy, burr hole craniotomy, and craniotomy. In these approaches of treatment, craniotomy was associated with the highest morbidity and largest area of surgical incision than the other two; twist drill craniotomy could have the smallest area of the surgical incision; however, it has the highest recurrence rates; burr hole craniotomy could have the lowest recurrence rates; however, it has the highest complication rates^5–9^. Meanwhile, the cost-effectiveness of the current treatment approaches was also unclear. Therefore, treatments for cSDH vary in different studies and hospitals^10^. Although these conventional treatments are primarily used and have advantages and disadvantages, their common problem is the relatively high recurrence rate. The recurrence rate of these techniques ranges from 2 to 37%, with most estimates being from 10 to 20%^3^. Therefore, new treatment techniques to further reduce the recurrence rate and surgical injury and improve the cure rate and quality of life of patients are significantly necessary.

Middle meningeal artery embolization (MMAE) provides a new therapeutic approach to the treatment of cSDH. As an interventional embolization treatment, MMAE could have a smaller surgical injury than conventional treatment, further reducing the complication and improving the prognosis. From a pathophysiological view, cSDH may be caused by continuous bleeding from the middle meningeal artery. Conventional treatments aimed to remove the hematoma but not the source of bleeding, which was one reason for the high recurrence rate^11,12^. MMAE achieves the therapeutic purpose by embolizing the middle meningeal artery, which theoretically can reduce the recurrence rate^13^. However, controversial results have been reported for MMAE compared to conventional treatment^14,15^. As a novel treatment, most previous studies had only a limited number of patients or were single-center series^14–18^. Moreover, Catapano *et al*.^19^ reported that although MMAE could have more surgery-related costs, its total 1-year costs could be lower due to its lower recurrence rate than conventional treatment. However, the cost-effectiveness of MMAE remains unclear. Therefore, this study aimed to (1) compare the functional outcomes, costs, and retreatment rates between patients with cSDHs undergoing MMAE and conventional treatment in the patients with cSDHs and (2) assess whether the MMAE is cost-effective compared to conventional treatment to evaluate the lifetime cost-effectiveness of MMAE from the perspective of healthcare payers in the United States.

## Materials and methods

### Study population

Patients from the Nationwide Readmissions Database (NRD) from 2016 to 2020 were reviewed. The NRD is supported by the Healthcare Cost and Utilization Project (HCUP, pronounced ʻH-Cupʼ). HUCP is a family of healthcare databases and related software tools and products developed through a Federal-State-Industry partnership and sponsored by the Agency for Healthcare Research and Quality (AHRQ) of the United States. The NRD is a unique and powerful database designed to support various types of analyses of national readmissions for all patients across the United States. The large sample size of the data with appropriate weights allows for national cross-sectional analyses. Demographic characteristics, hospital and regional information, diagnoses, procedures, discharge diagnoses, and readmission information were available. In addition, the NRD database included the total charge of each patient’s hospital stay, which could further support cost or cost-benefit analysis. No approval from the institutional review board or patient consent was required for this study because the NRD is publicly available and contains no identifiable patient information. Detailed information is available at https://hcup-us.ahrq.gov/db/nation/nrd/nrddbdocumentation.jsp. The study has been reported in line with the STROCSS criteria^20^ (Supplemental Digital Content 1, http://links.lww.com/JS9/B198).

**Figure 1:**
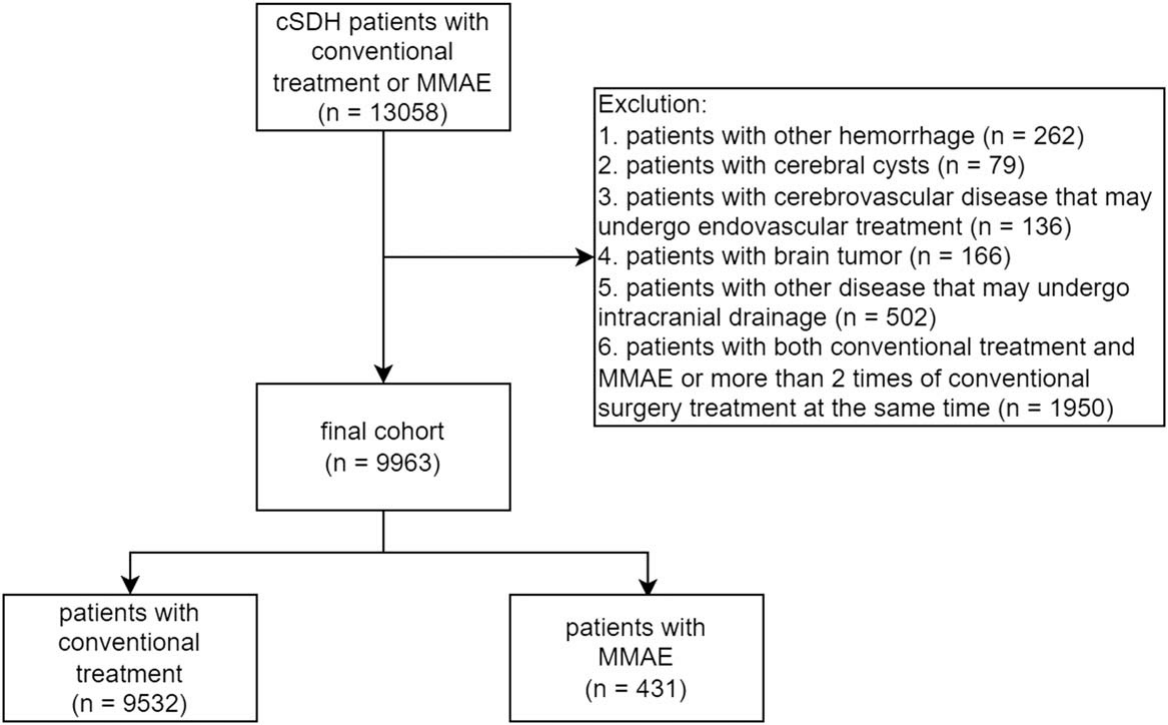
Flowchart of patient inclusion.

The International Classification of Diseases, 10th Revision, Clinical Modification (ICD-10-CM), and Procedure Codes (ICD-10-PCS) were used to select the patients. Patient’s first admission with a primary diagnosis of cSDH and who underwent conventional treatment or MMAE were included. Each patient was considered only once, and multiple admissions were not considered. The conventional treatment was defined as surgical management with hematoma removal, including twist drill craniotomy, burr hole craniotomy, and craniotomy. For patients with MMAE treatment, we excluded patients with cerebrovascular diseases possibly requiring endovascular treatment to avoid the patients’ misclassification. The total exclusion criteria were as follows: (1) patients with other types of hemorrhagic stroke (subarachnoid hemorrhage, intracranial hemorrhage, and extradural hemorrhage), (2) with cerebral cysts, (3) with cerebrovascular diseases possibly requiring endovascular treatment (including intracranial aneurysm, arteriovenous malformation, cavernous malformation, and arteriovenous fistula), (4) with brain tumors, (5) with other diseases possibly requiring intracranial drainage (brain abscess and hydrocephalus), and (6) with both conventional treatment and MMAE at the same time. The patient inclusion flowchart is shown in Figure 1. Detail codes of patients’ inclusions are illustrated in Supplementary Tables 1 (Supplemental Digital Content 1, http://links.lww.com/JS9/B199).

### Patient characteristics

Patients’ and hospitals’ baseline information was collected from the NRD database, including age, sex, elective admission (yes/no), primary payer (Medicare/nonMedicare), household income [0–50%/51–100% (0–100%: poorest to wealthiest population)], government control hospital (yes/no), large hospital bed size (yes/no), and teaching hospital (yes/no). All Patient Refined Diagnosis Related Groups risk (APRDRG) mortality scores (the degree of likelihood of death, including minor, moderate, major, and extreme) and severity scores (the degree of loss of function, including minor, moderate, major, and extreme) were collected. For the comorbidities, we used the Elixhauser Comorbidity Score^21^, which identifies 38 different pre-existing conditions based on diagnoses listed on hospital administrative data from the NRD database. In this study, we used the Readmission Elixhauser Comorbidity Index (risk of 30-day, all-cause readmission) and Mortality Elixhauser Comorbidity Index (risk of in-hospital mortality) provided by the HCUP (version 2023.1) (https://hcup-us.ahrq.gov/toolssoftware/comorbidityicd10/comorbidity_icd10.jsp). Comorbidities used in the Elixhauser Comorbidity Index included acquired immune deficiency syndrome, alcohol abuse, anemias due to other nutritional deficiencies, autoimmune conditions, chronic blood loss anemia iron deficiency, leukemia, lymphoma, metastatic cancer, solid tumor without metastasis in situ, solid tumor without metastasis malignant, cerebrovascular disease, heart failure, coagulopathy, dementia, depression, diabetes with chronic complications, diabetes without chronic complications, drug abuse, hypertension complicated, hypertension uncomplicated, liver disease mild, liver disease moderate to severe, chronic pulmonary disease, neurological disorders affecting movement, other neurological disorders, seizures and epilepsy, obesity, paralysis, peripheral vascular disease, psychoses, pulmonary circulation disease, renal failure moderate, renal failure severe, hypothyroidism, other thyroid disorders, peptic ulcer disease with bleeding, valvular disease, and weight loss^22^. Moreover, we collected information on dyslipidemia, smoking, and long-term use of anticoagulants or antiplatelets. Adverse events included epilepsy, aphasia, cerebral edema, cerebral hernia, coma, dysphagia, cranial nerve palsy, diplopia, postprocedural fever, pressure ulcer, sepsis, hypo-osmolar hyponatremia, pulmonary embolism, acute deep vein thrombosis, periprocedural stroke, acute myocardial infarction, acute respiratory failure, and acute kidney injury. Details codes are illustrated in Supplementary Table 1 (Supplemental Digital Content 1, http://links.lww.com/JS9/B199).

### Comparative analysis

We divided the entire cohort into the conventional treatment group and the MMAE group. Since patients in different groups could have different baseline levels, we used propensity score matching (PSM) to minimize the covariate imbalance. The propensity score was calculated using multivariate logistic regression models, which were adjusted for all covariates, including all baseline characteristics, comorbidities, and adverse events collected in this study. For the Elixhauser Comorbidity Index, we adjust for all the comorbidities, but not the Readmission Elixhauser Comorbidity Index or Mortality Elixhauser Comorbidity Index. An optimal matching approach with a clipper of 0.01 was used. Categorical variables are presented as the total number and percentage and analyzed using Fisher’s exact or Pearson’s *χ* ^2^-test. However, continuous variables are presented as the mean value and SD and were analyzed using the Student’s *t*-test. All analyses were performed within a complex samples function, with discharge weights provided by the NRD to ultimately yield accurate national estimates. All analyses were performed using the R software. All tests were two-sided, and statistical significance was set at *P*<0.05.

### Outcomes of the comparative analysis

The outcomes were 90-day retreatment rate, functional outcome, hospitalization cost, and length of hospital stay. Retreatment was defined as undergoing conventional treatment and/or MMAE during readmission. Functional outcomes were evaluated by patient discharge disposition. This patient discharge disposition has a high concordance with the degree of disability 90 days after discharge^23^. Favorable outcomes were defined as discharge to home, self-care (with or without special services), or short-term rehabilitation. Poor functional outcomes were defined as discharge to a skilled nursing, intermediate, or long-term care facility, hostility to medical advice, and missing disposition data. In addition, in-hospital mortality data were also collected.

### Model of cost-effectiveness analysis

**Figure 2:**
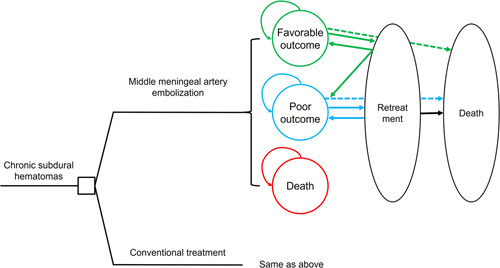
The schematic structure of the cost-effectiveness model for patients with chronic subdural hematomas. Patients enter the model to receive either middle meningeal artery embolization or conventional treatment. In the first 3 months, they move to a health state. Subsequently, the patients enter the 3-month cycles, in which they would transfer to death from other causes, stay in the same health state, or experience a retreatment.

After the comparative analysis, we conducted a cost-effectiveness analysis between the conventional treatment and MMAE groups. Based on the natural characteristics of cSDH treatment, we hypothesized that most patients would undergo retreatment within 90 days. After 90 days, patients who did not undergo retreatment would remain at risk of retreatment, but only at a relatively constant low risk^16^. Similar to the first treatment, we hypothesized that patients undergoing retreatment would still fit this profile. Based on these hypotheses, we developed a Markov model using TreeAge Pro 2023 (Williamstown, MA, USA) to evaluate the cost-effectiveness of MMAE versus conventional treatment. The structure of the model is illustrated in Figure 2. In the first 3 months, patients entered the model to receive either MMAE or conventional treatment and moved to one of the three possible health states based on the degree of disability. Subsequently, the patients received retreatment according to the first 90-day retreatment rate. After the first three months, a Markov state-transition model was developed to simulate disease progression until all patients reached 99 years old. Patients might remain in the same state, undergo retreatment, or die from other causes. The absorbing state was death, and the cycle length was 3 months.

### Costs, probabilities, and utilities of the cost-effectiveness analysis

This study was conducted from the healthcare payers’ perspective in the United States, and only direct costs were considered. The first and readmission hospitalization costs of different function outcomes were obtained from the comparative analysis in the current study. Annual posthospitalization costs of different function outcomes were obtained from a previous study^24^.

For the probabilities, the transition probabilities to different outcomes of the MMAE or conventional treatment during the first 3 months were extracted directly from the comparative analysis in the current study. The retreatment rates in the first three months were set as the 90-day retreatment rate in the present study, and the retreatment rates per three months (after the first 3 months) were set to one-tenth of the retreatment rates in the first three months. We hypothesized that conventional treatment is the only retreatment strategy for all the patients. Thus, the relative probabilities of retreatment were consistent with those observed for conventional treatment. The background age-specific death rate was derived from the most recent published census of US Life Tables^25^. The death hazard ratios for patients with poor outcomes compared with favorable outcomes were obtained from a previous study^26^.

The utilities of the different health states were measured using quality-adjusted life years (QALY) ranging from 0 to 1 and were assigned to all health states. A QALY of 1 means perfect health, and 0 means death. Utilities were obtained from a study by Hong and Saver^27^. Utilities of patients with favorable outcomes, poor functional outcomes, or who died are presented in Table 3. A discount rate of 3% annually was applied to both costs and utilities^28^.

### Measuring cost-effectiveness

The primary measure was the incremental cost-effectiveness ratio (ICER). ICER was calculated as the incremental cost per additional QALYs gained. The ICER was assessed using the recommended willingness-to-pay (WTP) threshold in the USA of $100 000 per QALY^29^.

### Sensitivity analysis of cost-effectiveness

Probabilistic sensitivity analysis (PSA) was also performed, with all parameters varying simultaneously. In total, 10 000 iterations were performed to evaluate the effects of the uncertainty. A cost-effectiveness acceptability curve based on the results of 10 000 iterations was generated to evaluate the cost-effectiveness likelihood of different strategies at different WTP thresholds. Moreover, key variables, including retreatment rates in the first three months, retreatment rates per 3 months after the first three months, and probabilities of favorable outcomes after different treatments were entered in one-way, two-way, and probabilistic sensitivity analyses over a wide range.

## Results

### Baseline information

We reviewed the 13 058 patients before the exclusion criteria. As the exclusion criteria, we excluded 262 patients with other hemorrhagic diseases, 79 with cerebral cysts, 136 with cerebrovascular diseases requiring endovascular treatment, 166 with brain tumors, 502 with diseases requiring intracranial drainage, and 1950 with both conventional treatment and MMAE or more than two times of conventional surgery treatment at the same time (Fig. 1). Our final analyses enrolled 9963 patients, including 9532 (95.7%) who received conventional treatment, and 431 (4.3%) who underwent MMAE. The mean age of the total cohort was 70.21±15.55 years, and 27.2% of the patients were female. Most patients had a minor or moderate APRDRG mortality score (57.0%) and a severity score (74.1%). The mean Readmission and Mortality Elixhauser score was 7.78±7.56 and 21.06±15.60, respectively. In general, patients treated for MMAE were more likely to be electively admitted (43.5 vs. 16.4%, *P*<0.001) in a large hospital (83.3 vs. 69.7%, *P*<0.001). Patients treated for MMAE had a higher prevalence of minor and moderate APRDRG mortality and severity score, lower Mortality Elixhauser score (16.66±16.22 vs. 21.26±15.55, *P* <0.001), a lower prevalence of aphasia (6.4 vs. 14.6%, *P*<0.001), cerebral edema (3.4 vs. 7.8%, *P*=0.008) and cerebral hernia (20.8 vs. 42.4%, *P*<0.001) and a higher prevalence of acute deep vein thrombosis (4.6 vs. 2.0%, *P*=0.008) (Table 1).

**Table 1:** Baseline information in the original cohort and PSM cohort.

	MMAE (*n*=431)	Conventional treatment (*n*=9532)	*P*	MMAE (*n*=431)	Conventional treatment (*n*=521)	*P*
Demographic information						
Age [mean (SD)]	71.0 (11.6)	70.2 (15.7)	0.298^a^	71.0 (11.6)	72.3 (15.9)	0.397^a^
Female (%)	93 (21.6)	2597 (27.2)	0.047	93 (21.6)	129 (24.7)	0.414
Government control hospital (%)	336 (77.9)	8178 (85.8)	0.001	336 (77.9)	421 (80.8)	0.436
Large hospital bed size (%)	359 (83.3)	6643 (69.7)	<0.001	359 (83.3)	405 (77.8)	0.17
Teaching hospital (%)	429 (99.5)	9256 (97.1)	0.032	429 (99.5)	517 (99.4)	0.847
Medicare payment (%)	127 (29.6)	2882 (30.2)	0.834	127 (29.6)	144 (27.6)	0.651
>50% family income (%)	246 (57.1)	4138 (43.4)	0.001	246 (57.1)	283 (54.4)	0.578
Elective admission (%)	187 (43.5)	1559 (16.4)	<0.001	187 (43.5)	214 (41.1)	0.547
Score systems						
APRDRG risk mortality score (%)			<0.001			0.977
Minor	191 (44.3)	2886 (30.3)		191 (44.3)	234 (45.0)	
Moderate	118 (27.4)	2483 (26.1)		118 (27.4)	134 (25.7)	
Severe	91 (21.1)	3133 (32.9)		91 (21.1)	112 (21.5)	
Extreme	31 (7.3)	1030 (10.8)		31 (7.3)	41 (7.9)	
APRDRG severity score (%)			0.003			0.8
Minor	160 (37.0)	4261 (44.7)		160 (37.0)	182 (35.0)	
Moderate	1667 (38.7)	2789 (29.3)		1667 (38.7)	192 (36.9)	
Severe	73 (16.9)	1398 (14.7)		73 (16.9)	107 (20.5)	
Extreme	31 (7.3)	1084 (11.4)		31 (7.3)	40 (7.6)	
Comorbidities						
AHRQ readmission Elixhauser score (mean (SD))	8.02 (8.06)	7.77 (7.54)	0.642^a^	8.02 (8.06)	7.92 (8.05)	0.891^a^
AHRQ mortality Elixhauser score (mean (SD))	16.66 (16.22)	21.26 (15.55)	<0.001^a^	16.66 (16.22)	16.72 (16.26)	0.966^a^
Acquired immune deficiency syndrome (%)	2 (0.4)	64 (0.7)	0.544^b^	2 (0.4)	2 (0.4)	0.968^b^
Alcohol abuse (%)	27 (6.3)	626 (6.6)	0.886	27 (6.3)	20 (3.9)	0.205
Anemias due to other nutritional deficiencies (%)	61 (14.0)	1389 (14.6)	0.824	61 (14.0)	73 (14.1)	0.999
Autoimmune conditions (%)	13 (3.0)	283 (3.0)	0.999	13 (3.0)	11 (2.2)	0.573
Chronic blood loss anemia iron deficiency (%)	4 (0.8)	44 (0.5)	0.432^b^	4 (0.8)	5 (1.0)	0.834^b^
Leukemia (%)	7 (1.6)	105 (1.1)	0.400	7 (1.6)	8 (1.5)	0.872
Lymphoma (%)	4 (1.0)	70 (0.7)	0.613^b^	4 (1.0)	3 (0.5)	0.481^b^
Metastatic cancer (%)	7 (1.6)	99 (1.0)	0.421	7 (1.6)	14 (2.7)	0.479
Solid tumor without metastasis in situ (%)	0 (0.0)	1 (0.0)	0.832^b^	0 (0.0)	0 (0.0)	–
Solid tumor without metastasis malignant (%)	20 (4.5)	249 (2.6)	0.068	20 (4.5)	16.6 (3.2)	0.398
Cerebrovascular disease (%)	431 (100.0)	9532 (100.0)	–	431 (100.0)	521 (100.0)	–
Heart failure (%)	53 (12.2)	967 (10.1)	0.294	53 (12.2)	72 (13.9)	0.605
Coagulopathy (%)	46 (10.7)	805 (8.4)	0.237	46 (10.7)	47 (9.1)	0.536
Dementia (%)	47 (10.8)	1181 (12.4)	0.464	47 (10.8)	72 (13.8)	0.316
Depression (%)	47 (10.9)	1063 (11.2)	0.923	47 (10.9)	49 (9.5)	0.618
Diabetes with chronic complications (%)	68 (15.7)	1404 (14.7)	0.670	68 (15.7)	87 (16.7)	0.801
Diabetes without chronic complications (%)	65 (15.0)	1404 (14.7)	0.908	65 (15.0)	83 (15.9)	0.796
Drug abuse (%)	6 (1.4)	106 (1.1)	0.695	6 (1.4)	9 (1.8)	0.684
Hypertension complicated (%)	98 (22.6)	1751 (18.4)	0.090	98 (22.6)	114 (21.9)	0.855
Hypertension uncomplicated (%)	212 (49.3)	5205 (54.6)	0.109	212 (49.3)	244 (46.9)	0.616
Liver disease mild (%)	20 (4.7)	333 (3.5)	0.325	20 (4.7)	19 (3.7)	0.554
Liver disease moderate to severe (%)	13 (2.9)	79 (0.8)	0.002	13 (2.9)	16 (3.2)	0.875
Chronic pulmonary disease (%)	71 (16.4)	1289 (13.5)	0.217	71 (16.4)	75 (14.3)	0.534
Neurological disorders affecting movement (%)	29 (6.8)	293 (3.1)	0.003	29 (6.8)	21 (4.0)	0.189
Other neurological disorders (%)	126 (29.2)	5277 (55.4)	<0.001	126 (29.2)	141 (27.0)	0.603
Seizures and epilepsy (%)	70 (16.2)	1264 (13.3)	0.201	70 (16.2)	87 (16.6)	0.901
Obesity (%)	18 (4.3)	715 (7.5)	0.071	18 (4.3)	20 (3.7)	0.777
Paralysis (%)	35 (8.1)	1979 (20.8)	<0.001	35 (8.1)	36 (6.9)	0.630
Peripheral vascular disease (%)	38 (8.8)	511 (5.4)	0.023	38 (8.8)	39 (7.4)	0.565
Psychoses (%)	9 (2.2)	257 (2.7)	0.584	9 (2.2)	12 (2.2)	0.960
Pulmonary circulation disease (%)	1 (0.3)	170 (1.8)	0.056^b^	1 (0.3)	0 (0.0)	0.272^b^
Renal failure moderate (%)	39 (9.1)	973 (10.2)	0.568	39 (9.1)	39 (7.6)	0.582
Renal failure severe (%)	26 (6.1)	334 (3.5)	0.035	26 (6.1)	32 (6.1)	0.992
Hypothyroidism (%)	68 (15.7)	1175 (12.3)	0.135	68 (15.7)	56 (10.8)	0.111
Other thyroid disorders (%)	1 (0.3)	93 (1.0)	0.170^b^	1 (0.3)	2 (0.5)	0.692^b^
Peptic ulcer disease x bleeding (%)	3 (0.8)	46 (0.5)	0.557^b^	3 (0.8)	10 (1.8)	0.287^b^
Valvular disease (%)	31 (7.3)	551 (5.8)	0.319	31 (7.3)	41 (7.9)	0.792
Weight loss (%)	20 (4.7)	555 (5.8)	0.457	20 (4.7)	34 (6.5)	0.376
Dyslipidemia (%)	217 (50.3)	4190 (44.0)	0.053	217 (50.3)	248 (47.6)	0.565
Anticoagulant and antiplatelet use (%)	125 (28.9)	2381 (25.0)	0.169	125 (28.9)	129 (24.8)	0.315
Smoking (%)	31 (7.2)	1054 (11.1)	0.058	31 (7.2)	30 (5.7)	0.508
Adverse events						
Epilepsy (%)	66 (15.4)	1564 (16.4)	0.668	66 (15.4)	85 (16.3)	0.794
Aphasia (%)	28 (6.4)	1392 (14.6)	<0.001	28 (6.4)	37 (7.1)	0.786
Cerebral edema (%)	15 (3.4)	744 (7.8)	0.008	15 (3.4)	22 (4.2)	0.649
Cerebral hernia (%)	90 (20.8)	4039 (42.4)	<0.001	90 (20.8)	104 (19.9)	0.810
Coma (%)	38 (8.7)	1127 (11.8)	0.121	38 (8.7)	44 (8.5)	0.927
Dysphagia (%)	19 (4.5)	612 (6.4)	0.194	19 (4.5)	26 (5.0)	0.785
Cranial nerve palsy (%)	0 (0.0)	37 (0.4)	0.444^b^	0 (0.0)	0 (0.0)	–
Diplopia (%)	2 (0.4)	40 (0.4)	0.889^b^	2 (0.4)	1 (0.2)	0.789^b^
Postprocedural fever (%)	0 (0.0)	16 (0.2)	0.541^b^	0 (0.0)	0 (0.0)	–
Pressure ulcer (%)	7 (1.5)	164 (1.7)	0.836	7 (1.5)	15 (2.9)	0.328
Sepsis (%)	3 (0.7)	135 (1.4)	0.355^b^	3 (0.7)	3 (0.5)	0.733^b^
Hypo-osmolar hyponatremia (%)	29 (6.7)	828 (8.7)	0.309	29 (6.7)	34 (6.6)	0.954
Pulmonary embolism (%)	3 (0.6)	64 (0.7)	0.844^b^	3 (0.6)	6 (1.1)	0.500^b^
Acute deep vein thrombosis (%)	20 (4.6)	189 (2.0)	0.008	20 (4.6)	20 (3.7)	0.652
Periprocedural stroke (%)	0 (0.0)	2 (0.0)	0.832^b^	0 (0.0)	0 (0.0)	–
Acute myocardial infarction (%)	1 (0.3)	57 (0.6)	0.463^b^	1 (0.3)	2 (0.4)	0.841^b^
Acute respiratory failure (%)	14 (3.2)	558 (5.9)	0.095	14 (3.2)	7 (1.3)	0.147
Acute kidney injury (%)	28 (6.4)	697 (7.3)	0.609	28 (6.4)	43 (8.2)	0.481

a Student’s *t*-test.

b Fisher’s exact test; All others were Pearson *χ* ^2^test.

APRDRG, All Patient Refined Diagnosis Related Groups; MMAE, middle meningeal artery embolization; PSM, propensity score matching.

### Comparison between MMAE and conventional treatment

After the PSM, all characteristics of patient baseline, hospital baseline, comorbidities, and adverse events showed no statistical differences (Table 1). In the PSM analysis, patients undergoing MMAE had a higher cost (US$11 9757.71±90 378.70 vs. US$75 745.55±100 701.28, *P*<0.001), lower 90-day retreatment rate (2.6 vs. 9.0%, *P*=0.001), and a shorter length of hospital stay (4.61±6.19 vs. 5.730±5.76 days, *P*=0.037) compared with those receiving conventional treatment (Table 2). These results were in line with the original cohort before PSM. In the original cohort, patients undergoing MMAE had more favorable outcomes than those receiving conventional treatment (80.9 vs. 69.7%, *P*=0.004). However, in the PSM cohort, although patients undergoing MMAE had a higher proportion of favorable outcomes (80.9 vs. 74.8%, *P*=0.224), the difference between the two groups was not statistically significant.

**Table 2:** Outcomes of PSM.

Characteristics	MMAE	Conventional treatment	*P*
Outcomes at discharge (%)			0.224
Favorable outcome	348 (80.9)	389 (74.8)	
Poor outcome	78 (18.0)	129 (24.5)	
Mortality	5 (1.1)	3 (0.7)	
Length of hospital stay [days, mean (SD)]	4.61 (6.19)	5.73 (5.76)	0.037^a^
Retreatment rate at the first 3 months (%)	11 (2.6)	47 (9.0)	0.001
Cost [US$, mean (SD)]	119 757.71 (90378.70)	75 745.55 (100701.28)	<0.001^a^
Favorable outcome	103 665.73 (71738.29)	62 767.05 (64039.56)	
Poor outcome	187 852.75 (127205.61)	113 755.15 (163725.86)	
Mortality	196 399.66 (95986.05)	106 737.04 (25660.53)	

a Student’s *t*-test; All others were Pearson *χ* ^2^-test.

MMAE, middle meningeal artery embolization; PSM, propensity score matching.

### Results of the cost-effectiveness analysis

**Figure 3:**
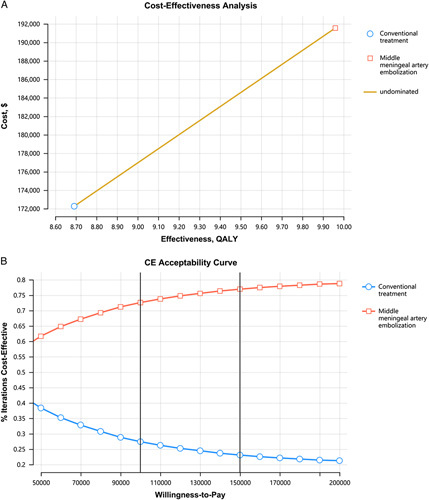
Cost-effectiveness and cost-effectiveness acceptability curves between the middle meningeal artery embolization and conventional treatment. A, incremental cost-effectiveness ratio curve; B, cost-effectiveness acceptability curve.

The inputs of the cost-effectiveness analysis are shown in Table 3. The results of the base-case analysis are presented in Table 4. MMAE was associated with an additional cost of US$19 280.0 with an additional QALY of 1.3 over a lifetime compared with conventional treatment, and its ICER was US$15 199.8/QALY. In PSA, patients treated with MMAE had a 72.6–76.9% probability of cost-effectiveness under the WTP threshold of US$100 000/QALY to US$150 000/QALY (Fig. 3).

**Table 3:** Input parameters of cost-effectiveness analysis.

Characteristics	Base-case value	Distribution	Range	Source
Start age	71	–	60–80	^a^
Cost (US$)				
Conventional treatment with favorable outcomes	62 767.05	Gamma, SD 64 039.56	47 075.29-48 458.82	^a^
Conventional treatment with poor outcomes	113 755.15	Gamma, SD 163 725.86	85 316.36-142 193.94	^a^
Conventional treatment with death	106 737.04	Gamma, SD 25 660.53	80058.78-133 421.30	^a^
MMAE with favorable outcomes	103665.73	Gamma, SD 71 738.29	77749.30-129 582.17	^a^
MMAE with poor outcomes	187 852.75	Gamma, SD 127 205.61	140889.56-234 815.94	^a^
MMAE with death	196 399.66	Gamma, SD 95 986.05	147299.75-245 499.58	^a^
Annual posthospitalization after favorable outcomes	4718.00	Gamma, SD 2359.00	3538.50-5897.50	^24^
Annual posthospitalization after poor outcomes	12 085.00	Gamma, SD 6042.5	9063.75-15 106.25	^24^
Probabilities of outcome at the first 3 mo				^a^
Favorable outcomes for patients undergoing conventional treatment	74.8%	Beta, SD 10%	67.32–82.28%	^a^
Death for patients undergoing conventional treatment	0.7%	Beta, SD 10%	0.63–0.77%	^a^
Poor outcomes for patients undergoing conventional treatment	24.5%	Calculated as 1- (probability of favorable outcomes + probability of death)	^a^	
Favorable outcomes for patients undergoing MMAE	80.9%	Beta, SD 10%	72.81–88.99%	^a^
Death for patients undergoing MMAE	1.1%	Beta, SD 10%	0.99–1.21%	^a^
Poor outcomes for patients undergoing MMAE	18.0%	Calculated as 1- (probability of favorable outcomes + probability of death)	^a^	
Probabilities of retreatment rate at the first 3 months				
Retreatment rate for patients receiving conventional treatment	9.00%	Beta, SD 10%	0–40.00%	^a^
Retreatment rate for patients undergoing MMAE	2.60%	Beta, SD 10%	0–10.00%	^a^
Probabilities of retreatment rate after the first 3 months				
Retreatment rate for patients receiving conventional treatment	0.90%	Beta, SD 10%	0–4.00%	^a^
Retreatment rate for patients undergoing MMAE	0.26%	Beta, SD 10%	0–1.00%	^a^
Age-specific death rate	US life tables	–	–	^25^
Death hazard ratios for patients with poor outcomes (compared with favorable outcomes)	8.310	Lognormal	5.480–12.581	^26^
Utility				^27^
Favorable outcomes	0.906	Beta, SD 10%	0.65–1.00	
Poor outcomes	0.319	Beta, SD 10%	0.15–0.50	
Death	0	–	–	
Discount rate	0.030	–	–	^28^

a Data were extracted from the PSM cohort.

MMAE, middle meningeal artery embolization.

**Table 4:** Base-case cost-effectiveness analysis.

Strategy	Cost	QALY	ICER
MMAE	191 538.3	10.0	15 199.8
Conventional treatment	172 258.3	8.7	
Difference	19 280.0	1.3	

INER, incremental cost-effectiveness ratio; MMAE, middle meningeal artery embolization; QALY, quality-adjusted life years.

**Figure 4:**
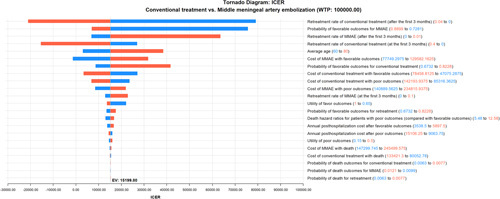
Tornado diagram depicting results of one-way sensitivity analyses in the comparison between patients treated with middle meningeal artery embolization and conventional treatment alone. The diagram shows how the higher and lower values of a single parameter affect the incremental cost-effectiveness ratio.

**Figure 5:**
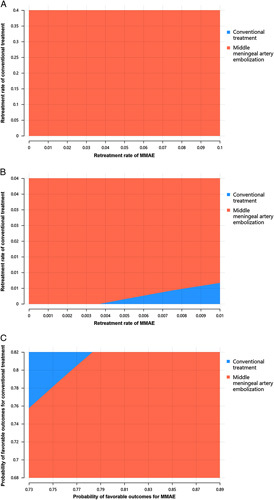
Two-way sensitivity analyses in the comparison between patients treated with middle meningeal artery embolization alone and conventional treatment alone and between combined treatment and conventional treatment alone. A, retreatment rates of MMAE and conventional treatment at the first 3 months; B, retreatment rates of MMAE and conventional treatment after the first 3 months; C, probabilities of favorable outcomes of MMAE and conventional treatment.

The results of the one-way sensitivity analysis are presented in the tornado diagram (Fig. 4). The ICER was particularly sensitive to the retreatment rate of conventional treatment after the first three months, the probability of favorable outcomes for patients treated with the MMAE, the retreatment rate of the MMAE after the first 3 months, and the first 3 months’ retreatment rate of conventional treatment. These parameters were further enrolled in the two-way sensitive analyses. The results showed that the MMAE had the dominant cost-effectiveness when the first 3 months retreatment rate was between 0 and 10% and the first 3 months of conventional treatment between 0 and 40% (Fig. 5A). The MMAE was the most cost-effective treatment in large scale of retreatment rates after the first 3 months and the probability of favorable outcomes (Fig. 5B and C).

## Discussion

In this study, we demonstrated that patients with cSDH undergoing MMAE had lower 90-day readmission rates and shorter lengths of hospital stay than those receiving conventional treatment. Although not statistically significant, patients undergoing MMAE alone had better functional outcomes than those receiving conventional treatment. Moreover, in the cost-effectiveness analyses, MMAE had higher quality-adjust life years by 1.3 over a lifetime, which were nearly 16 months of perfect health at excellent values, compared with conventional treatment. In the USA, compared with conventional treatment, MMAE yielded incremental cost-effectiveness ratios of $15 199.8/quality-adjust life year. To our knowledge, this study was the largest comparative study and the first cost-effectiveness analysis based on real-world data between MMAE and conventional treatment.

The treatment of cSDH by MMAE is currently one of the most discussed studies in this area. MMAE provides a new therapeutic perspective for patients with cSDH. Unlike previous treatments, MMAE mainly focuses on blocking the continuous formation of cSDH from a pathophysiological perspective. Thus, in theory, MMAE can potentially reduce the recurrence rates of cSDH. Some studies have reported satisfactory outcomes with MMAE. Recently, Kan *et al*. found that 70.8% of patients had greater than 50% improvement in imaging and that only 6.5% required further cSDH treatment in a perspective observation study^13,30^. However, they did not include patients receiving conventional treatment. Unfortunately, studies comparing MMAE with conventional treatment have been limited to a single-center or a small sample size. The results of these researches were also inconsistent^14–18^. Ban *et al*.^15^ found that MMAE had a lower treatment failure rate than conventional treatment. Ng *et al*. and Enriquez-Marulanda *et al*. found that MMAE had a lower recurrence rate (or a trend of lower recurrence rate)^14,17^, however, which was not shown in the results of the statistical analysis of the study of Matsumoto *et al.* ^16^. Therefore, in the first part, we compare the recurrence rate and clinical prognosis between the MMAE and conventional treatment using a multicenter, nationwide database to further analyses the differences between the MMAE and conventional treatment. Our results showed that MMAE had a lower 90-day retreatment rate, a shorter length of hospital stay, and similar functional outcomes compared to conventional treatment. These results are consistent with the anatomical and pathophysiological basis for MMAE treatment proposed by previous studies^31^. Our results of MMAE treatment may change the current consensus on the treatment of cSDH.

In our study, although the MMAE showed some advantages in the recurrence rate and clinical prognosis, it also increased the costs. Although the recurrence rate and clinical prognosis have been previously discussed, the cost-effectiveness of MMAE compared to conventional treatment is still unclear. Therefore, in the second part, we analyze whether the MMAE is cost-effective compared to conventional treatment. Previous studies had only focused on costs. Catapano *et al*. found that the MMAE has a higher first hospitalization cost but a lower total 1-year cost compared with conventional treatment. This was mainly because of the fewer unexpected additional treatments in MMAE^7^. Mira *et al*. found that the MMAE had a higher direct procedural cost but a similar overall cost in the first hospitalization compared with conventional treatment^8^. These studies were well executed but only considered short-term costs (one year or only in the first hospitalization) and did not consider the difference in benefits between the MMAE and conventional treatment.

Our study is based on a cost-effectiveness analysis, which considers the costs and benefits for different patients’ conditions over a lifetime. Different from the results of the study of Catapano *et al*., we found that the patients with MMAE not only had a higher hospitalization cost, but also had higher living costs. This result means that MMAE is still more expensive than conventional treatment in the long run. In the benefits analysis, MMAE had higher quality-adjust life years compared with conventional treatment. When considering the benefits, we found that the increased cost is acceptable compared to the increased benefits. In the USA, compared with conventional treatment, MMAE was cost-effective under the current US willingness-to-pay threshold. These results mean that the cost increase should not hinder the application and generalization of MMAE. From an economic point of view, the additional benefit of MMAE is greater than its additional cost.

Our study has limitations. First, owing to the retrospective review of administrative data, we were unable to obtain more granular data. Second, the treatment methods were determined mainly based on the ICD-10-PCS code. To minimize misclassification, we excluded patients with other diseases possibly requiring endovascular, drainage, or surgical treatment of the brain and head. However, a potential selection bias should still be considered. Third, as a new treatment approach, the application of MMAE tended to be specific to certain patients. Although we performed PSM to ameliorate the differences in comorbidities and clinical severity and generate more suitable comparison groups, it did not address the selection bias inherent to the database analysis. Fourth, in the absence of relevant data, we assumed that the probability of retreatment after the first 3 months was one-tenth of the retreatment rate in the first 3 months. These rates are important for cost-effectiveness analyses. Therefore, we performed a wide range of sensitivity analyses to test the stability of our results. Finally, although the MMAE could have a better treatment outcome than conventional treatment, it cannot immediately reduce hematoma volume to reduce the mass effect. Therefore, conventional treatment was still necessary for patients immediately after the removal of a hematoma volume. The current study’s results were unsuitable for this patient, and further studies are needed.

## Conclusion

In summary, patients with cSDH treated with MMAE had a lower 90-day retreatment rate, a shorter length of hospital stay, and similar functional outcomes than those treated with conventional treatment. Although MMAE had higher initial hospitalization costs, they were likely more cost-effective than conventional treatment. These comparative and cost-effectiveness analyses further support the consideration of a paradigm shift in cSDH treatment.

## Ethical approval

No approval from the institutional review board or patient consent was required for this study because the Nationwide Readmissions Database is publicly available and contains no identifiable patient information.

## Consent

None.

## Sources of funding

This work was supported by the National High Level Hospital Clinical Research Funding (BJ-2021-234) and Natural Science Foundation of China (81771233,82171290).

## Author contribution

P.Q. and A.L. conceived and designed the study; X.T. and X.X. analyzed the data and wrote the paper. All authors read and approved the final manuscript.

## Conflicts of interest disclosure

None.

## Research registration unique identifying number (UIN)

Name of the registry: not applicable.
Unique identifying number or registration ID: not applicable.
Hyperlink to your specific registration (must be publicly accessible and will be checked): not applicable.

## Guarantor

Aihua Liu.

## Availability of data and materials

The supporting data of this study are available on https://hcup-us.ahrq.gov.

## Provenance and peer review

Not commissioned, externally peer-reviewed.

## Supplementary Material

SUPPLEMENTARY MATERIAL
