# Supplementary material for: Comparative study on clinical outcomes and cost-effectiveness of chronic subdural hematomas treated by middle meningeal artery embolization and conventional treatment: a national cross-sectional study
Source: Int J Surg. 2023 Oct 12;109(12):3836–47. doi: 10.1097/JS9.0000000000000699 (PMC10720801; doi:10.1097/JS9.0000000000000699)
Supplement: SUPPLEMENTARY MATERIAL [file js9-109-3836-s002.docx]

Supplementary table 1 ICH-10-CM codes of characteristics used in the current study.

| Characteristics | ICD-10-CM codes or source |
| --- | --- |
| cSDH | I6203 |
| Subarachnoid hemorrhage | I60 |
| Intracranial hemorrhage | I629 |
| Extradural hemorrhage | I621 |
| Cerebral cysts | G930 |
| Intracranial aneurysm | I671 |
| Arteriovenous malformation | Q282 |
| Cavernous malformation | Q283 |
| Arteriovenous fistula | I77 |
| Brain tumor | C70, C71, C72, C32, C33, C793, C7931, C7932 |
| Brain abscess | G06, G07 |
| Hydrocephalus | G91 |
| Elixhauser score and comorbidity details | https://hcup-us.ahrq.gov/toolssoftware/comorbidityicd10/comorbidity_icd10.jsp |
| Dyslipidemia | E78 |
| Anticoagulant/antiplatelet use | Z790, Z7982 |
| Smoking | Z720, F172 |
| Epilepsy | R40, R56 |
| Aphasia | R47, I69320, I69920, I69120, I69020 |
| Cerebral edema | G936 |
| Cerebral hernia | G935 |
| Coma | R402, R403 |
